# Is Expansion a Cause of Leakage in Fillings?

**Published:** 1880-06

**Authors:** 


*Is Expansion a Cause of Leakage in Fillings?*—In conversation with an old and thoughtful dentist, the suggestion was made by him to the writer that one of the causes of the loss of the integrity of fillings was due to expansion; and he (a soft foil advocate,) was inclined to believe that one of the troubles with cohesive foil was that its density alone, making it more susceptible to thermal changes, caused the "starting" of such fillings, and their consequent leakage. The idea is of course not a new one, but it suggested to the writer the thought that some experiments might be made serviceable in determining this point; and associated with the cohesive and non-cohesive gold foils, experiments were made also upon tin foil and one or two varieties of amalgams.

A piece of ivory was selected of some size, and five holes drilled in it on the smooth side. These were made with great care, pains being taken that the sides should be parallel and as smooth as possible, so as to afford no obstacle to the starting of the fillings subsequently inserted.

The bottoms were concave, being formed by a plug-finishing bur of egg shape.

The first cavity was filled with the "Boston (Frost) Dental Amalgam;" the second with the "Standard Alloy;" the third with tin foil; the fourth with Abbey's soft or non-cohesive gold; and the fifth with annealed, highly cohesive gold. The tin and Abbey's foil were put in with hand pressure, and the cohesive foil malleted in with a steel mallet, the ivory being tightly screwed in a vise. The amalgams were worked rather dry, although somewhat plastic. When these last were seemingly solidly set, some hours having elapsed, the respective fillings were dressed off perfectly smooth, no burnishing being done, and finished with a flat scotch stone.

The piece thus finished was then laid in the snow on a cold day in winter, and when thoroughly chilled was plunged into boiling water. This alternate freezing and thawing was repeated three times. An inspection of the fillings was then made.

The Frost Amalgam had started quite visibly and the finger nail caught readily against its edges. The Standard Alloy was quite decidedly projected from its cavity, rather more so than the other amalgam. The tin foil was so slightly raised as to be hardly visible to the eye without a magnifier, and the condition of the non-cohesive and cohesive gold fillings were much the same as the tin. There was little perceptible difference between the two varieties of gold foil; if anything the non-cohesive filling was projected from its cavity slightly more than the other.

From this experiment it might be concluded that little difference exists between the relative expansion and consequent "starting" of fillings made respectively of tin, non-cohesive and cohesive foil, between the thermal points of 32° F., and 212° F. But as single experiments are worth little, these facts are made public that some such lines of investigation may be taken up and developed into certainty. H.

